# Case report: Variants in the *ERCC4* gene as a rare cause of cerebellar ataxia with chorea

**DOI:** 10.3389/fgene.2023.1107460

**Published:** 2023-02-02

**Authors:** Joanna Kulikowska, Anna Jakubiuk-Tomaszuk, Małgorzata Rydzanicz, Rafał Płoski, Jan Kochanowicz, Alina Kulakowska, Katarzyna Kapica-Topczewska

**Affiliations:** ^1^ Departament of Neurology, Medical University of Bialystok, Białystok, Poland; ^2^ Medical Genetics Unit, Mastermed Medical Center, Białystok, Poland; ^3^ Department of Medical Genetics, Medical University of Warsaw, Warsaw, Poland

**Keywords:** *ERCC4*, gene variant, chorea, ataxia, brain atrophy, NER (nucleotide excision repair)

## Abstract

Variants in the *ERCC4* gene have been described to be associated with the following autosomal recessive diseases: xeroderma pigmentosum group F (XPF), xeroderma pigmentosum type F/Cockayne syndrome (XPF/CS), Fanconi anemia complementation group Q (FANCQ), and XFE progeroid syndrome (XFEPS). In this paper, we present a case of a 53-year-old Caucasian female patient with rare variants in the *ERCC4* gene. When she was 42 years old, falls and loss of balance occurred. At the age of 48, involuntary, uncoordinated movements of the upper limbs and head, tongue stereotypes (licking and extending movements), speech problems (dysarthria), memory deterioration, and hearing loss occurred. Since childhood, she has shown hypersensitivity to UV radiation. The neurological examination revealed chorea syndrome, cerebellar ataxia, dysarthria, and bilateral hearing loss. She has numerous pigmented lesions on the skin. Brain MRI demonstrated massive cortico-subcortical atrophy. The neuropsychological examination revealed dysfunctions in the executive domain in terms of attention, working memory, organizing, and planning activities. The genetic diagnostics was performed which excluded spinocerebellar ataxia types 1, 2, 3, 6, and 17, Huntington’s disease, and FMR1 premutation. In the genetic analysis of next-generation sequencing (NGS), two variants: c.2395C > T and c.1349G > A in the *ERCC4* gene were identified in a heterozygote configuration. So far, a few cases of *ERCC4* gene variants, which are associated with nucleotide excision repair pathways, have been described in connection with symptoms of cerebellar ataxia. In patients with ERCC4 biallelic variants, the adult neurological phenotype can sometimes be the first symptom and reason for access to genetic testing. The aforementioned case highlights the occurrence of rare genetic causes of progressive neurodegenerative diseases in adults, especially with the spectrum of autosomal recessive nucleotide excision repair pathway disorders (NERDs).

## 1 Introduction

Chorea and cerebellar ataxia are rare causes of progressive neurodegeneration, most often of genetic etiology. After excluding secondary causes, like drug-induced dyskinesia or Sydenham chorea, genetic diagnosis must be conducted. In the differential diagnosis, the most common hereditary causes should be taken into consideration: Huntington’s disease, neuroacanthocytosis, spinocerebellar ataxia (SCA) types 1, 2, 3, 6, and 17, and others were found to be associated with chorea (Burgunder, 2022). Increasingly available genetic diagnostics, especially genomic sequencing, allows the detection of rare causes of cerebellar ataxia with chorea. *ERCC4* gene variants have been described to be associated with the following autosomal recessive diseases: xeroderma pigmentosum group F (XPF) (Nabouli et al., 2021), xeroderma pigmentosum type F/Cockayne syndrome (XPF/CS) (Kashiyama et al., 2013), Fanconi anemia complementation group Q (FANCQ) (Bogliolo et al., 2013a), and XFE progeroid syndrome (XFEPS) (Mori et al., 2018). So far, a few cases of *ERCC4* gene variants in connection with symptoms of cerebellar ataxia have been described. The role of nucleotide excision repair (NER) variants is highlighted in connection with adult-onset neurodegeneration (Cordts et al., 2022). Various gene variants can cause multiple phenotypes. We present a case of a patient with *ERCC4* gene variants with the rare phenotype of progressive cerebellar ataxia, chorea, cognitive impairment, and sensory hearing loss with a history of skin photosensitivity.

## 2 Case report

**FIGURE 1:**
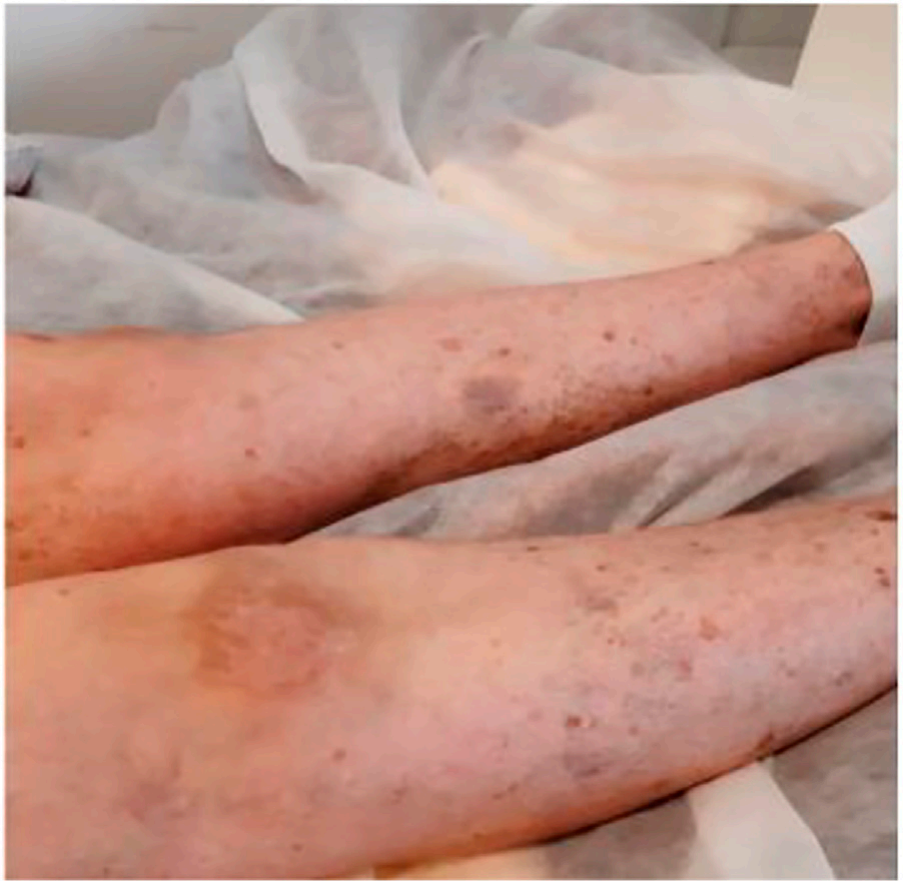
Hyperpigmentation lesions on lower limbs.

**FIGURE 2:**
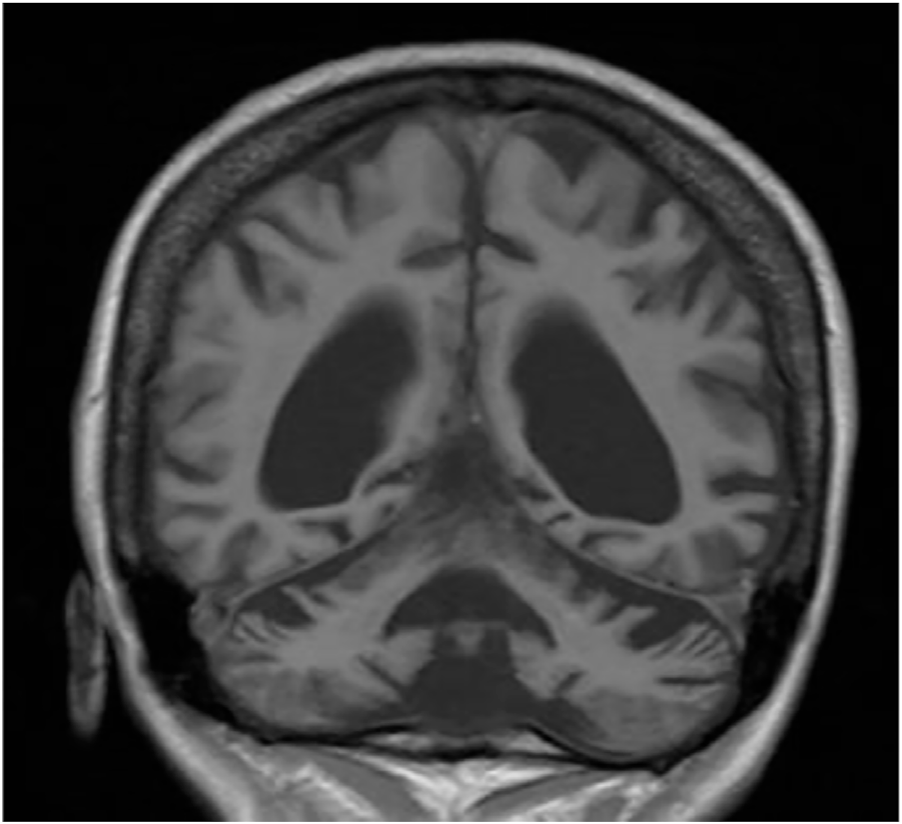
MRI—massive cortical and subcortical atrophy frontal section.

**FIGURE 3:**
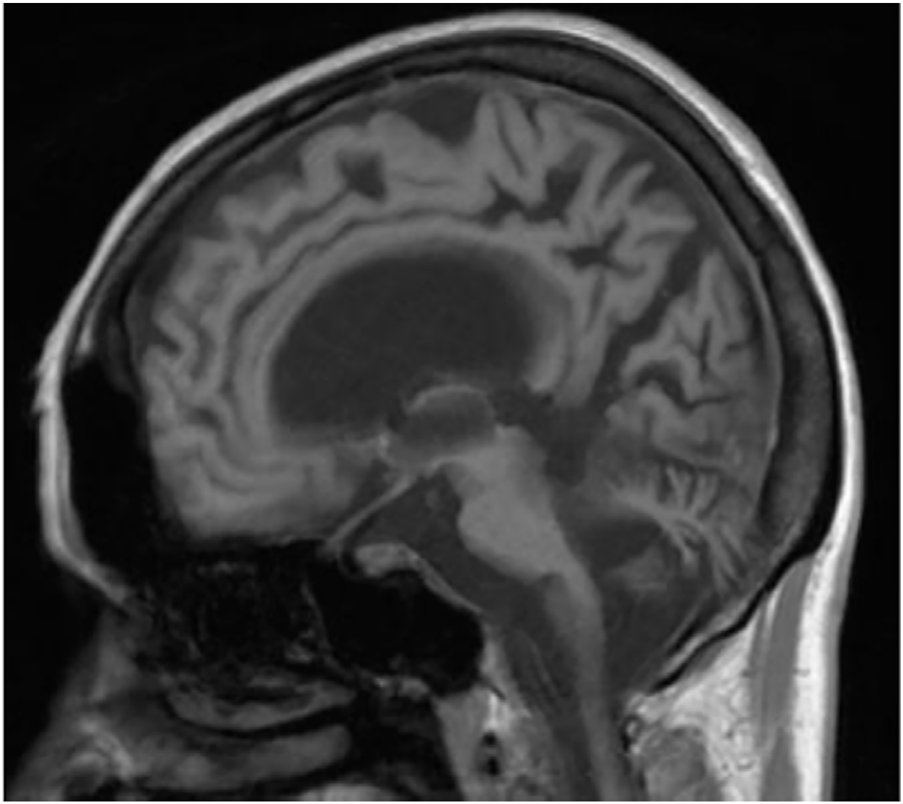
MRI—massive cortical and subcortical atrophy Sagittal section.

**FIGURE 4:**
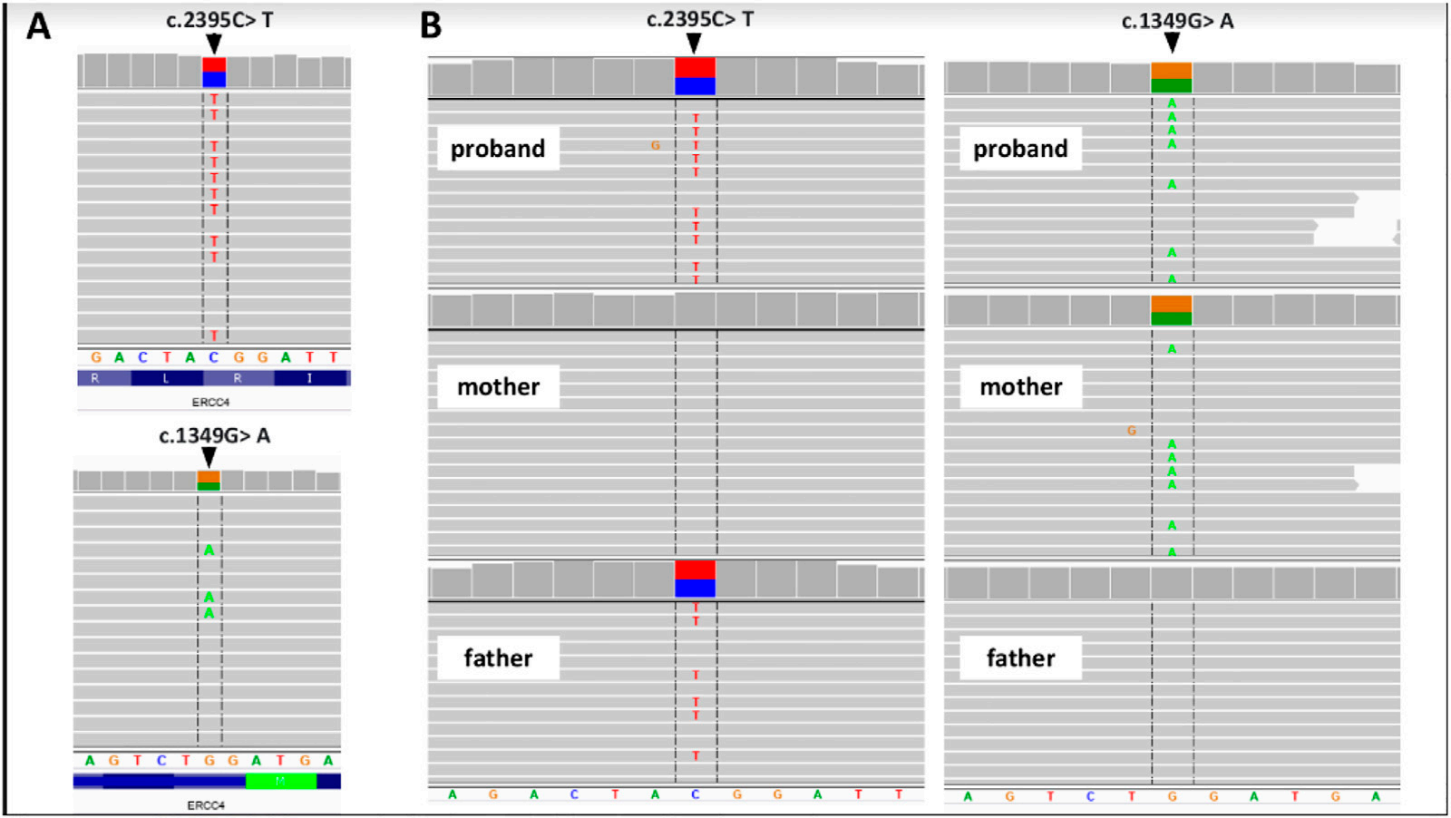
Genetic examination and family study of the ERCC4 gene. **(A)** Proband’s WES results and **(B)** results of family study performed by NGS-based amplicon deep sequencing. Integrative Genomics Viewer screenshots are presented.

We present a case of a 53-year-old Caucasian female patient. When she was 42 years old, falls and loss of balance occurred. Furthermore, at the age of 48, progressive, involuntary, uncoordinated movements of the upper limbs and head, tongue stereotypes (licking and extending movements), speech problems (dysarthria), memory deterioration, and hearing loss occurred. Since childhood, she has shown skin and conjunctiva hypersensitivity to the sun. She underwent splenectomy due to spherocytosis at the age of 18 and cholecystectomy at the age of 20. Before the age of 40, she was diagnosed with premature ovarian failure. *In vitro* fertilization was performed with a donor oocyte. She gave birth to a female baby. There was no family history of genetic diseases and consanguineous parents. She pursued philological education. Initially, she had been working as a teacher of the Russian language and then in a library. Currently, she is living on a pension. The neurological examination revealed chorea syndrome, cerebellar ataxia, dysarthria, and bilateral hearing loss. She has numerous pigmented lesions on her brown-colored skin (Figure 1). Brain MRI demonstrated massive cortico-subcortical atrophy (Figures 2, 3 ). The consulting otolaryngologist diagnosed bilateral sensory hearing impairment. The electroneurographic examination did not reveal any signs of peripheral polyneuropathy. Laboratory tests showed the presence of macrocytic anemia, significant vitamin B12 deficiency, and high levels of homocysteine. The consulting hematologist recommended supplementation with vitamin B12, iron, and folic acid. Endoscopic examination showed atrophic gastropathy. At the age of 48, a neuropsychological examination was performed which revealed cognitive-behavioral disorders with a predominance of subcortical symptomatology. Additionally, a slowdown in the pace of work, periodic drops in the tension of free attention, difficulties in organizing complex visual materials, and deficits in learning and memory, mainly of an executive nature, were described. The neuropsychological examination performed at the age of 53 revealed dysfunctions in the executive domain in the field of attention (maintenance, selectivity, and divisibility), working memory, organizing, and planning activities (mainly activities on visual materials). There was variability in the severity of dysfunction in the cognitive and non-cognitive spheres. The clinical details are presented in Table 1. The genetic diagnostics was performed which excluded spinocerebellar ataxia types 1, 2, 3, 6, and 17, Huntington’s disease, and FMR1 premutation. Exome sequencing using the next-generation sequencing (NGS; SureSelectXT Human All Exome v7 (Agilent)) method was performed in further genetic diagnostics as previously described (Jakubiuk-Tomaszuk et al., 2019). Two variants in the *ERCC4* gene, namely, missense c.2395C > T and a novel non-sense c.1349G > A, in the configuration of the heterozygote, were identified (Table 2). The presence of the c.1349G > A variant of the *ERCC4* gene was found in a heterozygous state in the proband’s mother but excluded in the father, while the presence of the c.2395C > T variant of the *ERCC4* gene was found in a heterozygous state in the proband’s father but excluded in the mother. It is consistent with *in trans* variant transmission in the autosomal recessive mode of inheritance. Moreover, in the proband, a heterozygous non-sense variant in the *SPTB* gene (hg38 chr14:064786837-C>T; NM_001355436.2:c.3128G>A (p.Trp1043Ter), rs1594775390) was identified (Figure 4). The c.3128G > A variant has 0 frequency in the gnomAD v3.1.2 database and was described in the ClinVar database as “pathogenic” and classified as “pathogenic” according to ACMG classification (total score 11, PVS1 very strong, PP5 moderate, and PM2 supporting). Variants in the *SPTB* gene, including the c.3128G > A variant identified in our patient, are associated with spherocytosis type 2, 8, and 9. The parents showed no clinical or laboratory signs of spherocytosis. So far, a rare phenotype of progressive spinocerebellar ataxia, chorea, cognitive impairment, and hearing loss with a history of skin photosensitivity has been described in single patients with *ERCC4* gene variants.

**TABLE 1:** Clinical details.

Personal information	—
Sex	Female
Age	53
Education	Master’s degree in philology
Child	Daughter after performing *in vitro* fertilization from a donor oocyte
Medical history	
Surgeries	Splenectomy due to spherocytosis (at the age of 18)
	Cholecystectomy (age 20)
History of cancer (including cutaneous cancer)	No
Fertility	Premature ovarian failure; *in vitro* fertilization was performed with a donor oocyte. She gave birth to a female baby
Symptoms	
First neurological symptoms (age)	42
First neurological symptoms	Falls and balance disorders
Current neurological symptoms	Involuntary, uncoordinated movements of the upper limbs and head, tongue stereotypes (licking and extending movements), speech problems (dysarthria), memory deterioration, and hearing loss
Sun hypersensitivity	Observed from childhood
Neurological assessment	
Current neurological examination abnormalities	Chorea syndrome, bilateral cerebellar syndrome, dysarthria, and bilateral hearing loss
Neuropsychological assessment	Dysfunctions in the executive domain in the field of attention, working memory, organizing, and planning activities
Additional tests	
Brain imaging	Massive cortico-subcortical atrophy was revealed in the supratentorial area
Electroneurography	No abnormalities
Abnormalities in laboratory tests	Macrocytic anemia, significant vitamin B12 deficiency, and high levels of homocysteine
Result of additional examination	Gastroscopy—atrophic gastropathy
Conducted specialist consultation	
Otolaryngological assessment	Bilateral hearing impairment
Hematology assessment	Supplementation with vitamin B12, iron, and folic acid
Ophthalmology assessment	No abnormalities

**TABLE 2:** Genetic diagnosis details.

c.DNA	Protein	Gene	Reference sequence	Chromosomal position hg38	Frequency in the gnomAD v3 database	Configuration
c.2395C>T	*p*. (Arg799Trp)	*ERCC4*	NM_005236.3	16:013947991-C > T	0.0007677	Heterozygous
c.1349G>A	*p*. (Trp450Ter)			16:013935281-G > A	0	Heterozygous
ACGM classification						

The c.3128G > A variant has 0 frequency in the gnomAD v3.1.2 database and is described in the ClinVar database as “pathogenic” and classified as “pathogenic” according to ACMG classification (total score 11, PVS1 very strong, PP5 moderate, and PM2 supporting).

## 3 Discussion

We present a case of a Caucasian patient with chorea syndrome and cerebellar ataxia. The genetic diagnostics was performed which excluded spinocerebellar ataxia type 1, 2, 3, 6, and 17, Huntington’s disease, and FMR1 premutation. Exome sequencing using the NGS method revealed two variants, c.2395C > T and c.1349G > A, in the *ERCC4* gene. NER is a mechanism responsible for DNA damage repair and occurs as a result of endogenous or exogenous factors (Spivak, 2015). NER impairment is associated with the occurrence of diseases such as XP and CS, and due to the possible occurrence of many variants of these genes, different phenotypes are described. Recently, attention has been paid to the development of adult-onset neurodegeneration in connection with damage to NER mechanisms, including the occurrence of *ERCC4* variants (Cordts et al., 2022). All of them presented progressive symptoms of neurodegeneration beginning in adulthood: ataxia, chorea, and global brain atrophy. The concept of nucleotide excision repair disorders (NERDs) has recently been introduced to cover the spectrum of patients with adult-onset neurodegeneration with diagnosed NER impairment. Cordts et al., 2022 described 13 patients with NER gene variants who presented progressive neurological disorders, of which eight patients showed variants in the *ERCC4* gene. In this study, it was estimated that among patients with ataxia, NER disorders occurred in 1% of patients and among patients with ataxia and cognitive disorders, NER disorders occurred in 8.6% (). *ERCC4* variants have been described to be associated with autosomal recessive inheritance diseases: XP (Nabouli et al., 2021), FANCQ (Bogliolo et al., 2013b), CS (Kashiyama et al., 2013), and XFEPS (Mori et al., 2018). XP is a disease characterized by hypersensitivity to the sun, the presence of sun-induced skin lesions, and a high risk of skin cancer development, usually in the first decade of life (Nabouli et al., 2021). Niraj et al. reported cases of two patients with XPF who showed signs of cerebellar ataxia, chorea syndrome, hearing loss, and global atrophy on brain MRI. One of these patients had a history of basal cell carcinoma of the skin. In both patients described in this report, the c.2395C > T variant in the *ERCC4* gene was identified (Shanbhag et al., 2018). Eight patients who carried variants in the *ERCC4* gene were reported with adult-onset neurological deterioration. Seven patients harbored missense c.2395C > T variant in the heterozygous state (Cordts et al., 2022). In our case, the second variant c.1349G> A has not been reported previously. Since childhood, our patient has been highly hypersensitive to solar radiation, but the oncological history remains negative. Doi et al. (2018) described the coexistence of slowly progressive cerebellar ataxia, chorea syndrome, and mild hypersensitivity to solar radiation, in association with ERCC4 gene mutation. The neurological symptoms of XP occur in 20–30% of cases, mainly in the XPF (OMIM # 278760). This phenotype is often classified as CS. CS (OMIM # 278760) is characterized by microcephaly, significant sensitivity to the sun, and progressive neurodegeneration from the first months of life. The mean age at death is 12.5 years. Despite the similar genetic etiology, the differences in the clinical picture, including the age of the first symptoms, do not allow the patient to be diagnosed with CS. In-depth neurological diagnostics of the presented patient, apart from the highly developed chorea syndrome and cerebellar ataxia, showed significant cognitive impairment. In addition, bilateral sensory hearing loss was detected. Imaging diagnostics showed generalized brain atrophy. It is worth noting that the patient suffered from spherocytosis as a child, due to which she underwent splenectomy. Currently, she is diagnosed with macrocytic anemia due to vitamin B12 deficiency. FA (OMIM #615272) is characterized by bone marrow failure, congenital anomalies, and possible sun hypersensitivity, and the patient usually dies in childhood. Apart from the abnormalities mentioned previously, no symptoms of bone marrow failure have been found in our patient so far. It is worth emphasizing that the family history remained negative. Variants in the *ERCC4* gene were found in the mother and father of the proband. In addition to increasing knowledge about NERD, the current literature does not provide a description of the coexistence of the two variants, c.2395 > T and c.1349G > A, in the *ERCC4* gene. Only a few cases of cerebellar ataxia have been described so far in connection with the variants of the *ERCC4* gene. The patient is aware of the genetic cause of her symptoms and has been informed about a very rare phenotype, which makes it impossible to attribute the symptoms to a specific genetic syndrome. However, due to the current cognitive impairment, information about the overall health condition is communicated to her husband. Adult-onset neurodegeneration, such as ataxia, chorea, cognitive impairment, and brain atrophy, should be considered a disrupted nucleotide excision repair mechanism, which has recently been referred to as NERD (Cordts et al., 2022). In patients with *ERCC4* biallelic variants, the adult neurological phenotype can sometimes be the first symptoms and reason for access to genetic testing. The aforementioned case highlights the occurrence of rare genetic causes of progressive neurodegenerative diseases in adults, especially with the spectrum of autosomal NERD.

## Data Availability

The original contributions presented in the study are included in the article/Supplementary Material. Further inquiries can be directed to the corresponding author.
